# SAKrificing an Essential Stress-Sensing Pathway Improves Aspergillus fumigatus Germination

**DOI:** 10.1128/msphere.00010-22

**Published:** 2022-01-26

**Authors:** Anaïs Hérivaux, Gustavo H. Goldman, Nicolas Papon

**Affiliations:** a Univ Angers, Univ Brest, IRF, SFR ICAT, Angers, France; b Faculdade de Ciências Farmacêuticas de Ribeirão Preto, Universidade de São Paulo, Ribeirão Preto, São Paulo, Brazil

**Keywords:** Aspergillus, lung infection, host-pathogen interaction, cell signaling, adaptation

## Abstract

Fungal infections represent a major problem in human health. This is particularly the case of infections caused by the filamentous fungus Aspergillus fumigatus, affecting millions of people worldwide. While active germination of conidia is documented to be essential for the A. fumigatus pathogenicity in the context of chronic infections, the molecular mechanisms underlying this morphogenetic transition remain unclear. In a new report, Kirkland and colleagues shed light on a central role of a major stress-sensing pathway in orchestrating the germination process in A. fumigatus. This work provides insight into disruption of an essential cell signaling circuitry for an adequate and long-term adaptation of the fungus to the lung microenvironment.

## COMMENTARY

Aspergillus fumigatus is a saprophytic mold which is ubiquitous in the environment. While major advances have been made in diagnostics and therapeutics, this deadly fungal species remains a major public health issue as it is responsible for a wide range of acute and chronic diseases, affecting several millions of people worldwide (1). In healthy people, inhaled fungal airborne conidia are actively cleared from the airways. Nevertheless, the locally altered immune defenses in immunocompromised patients and the deficient mucociliary clearance in patients with cystic fibrosis (CF) could result in invasive aspergillosis and chronic colonization of the lungs by A. fumigatus, respectively (2, 3).

Because of their small size (2 to 3 μm in diameter), the conidia can easily reach the alveoli in the lungs. Once embedded in the lung epithelium, conidia are known to actively germinate following well-defined steps: (i) the osmotic swelling of conidia to form germ tubes and (ii) the polarized growth of the germ tubes resulting in the development of hyphae (2, 3). Investigations have demonstrated that germination of conidia is only possible whether their environment is suitable (for instance, adequate nutrient availability and the presence of potential stressors). Unlike A. nidulans (another ubiquitous mold with low pathogenic potential) which is able to induce germination of conidia with glucose as the sole nutrient source, A. fumigatus also requires water and oxygen to trigger germination (4, 5). Importantly, *in vivo* experiments showed that A. fumigatus strains which are able to germinate rapidly are more virulent in the lung microenvironment compared to slow-germinating strains (6). Obviously, this led some research groups to decipher the molecular mechanisms governing the germination process in this pathogenic mold within the lung low-nutrient microenvironment (7). Such research could indeed potentially lead to the identification of new fungal targets for therapeutic development purposes. In this context, unprecedented insights into molecular regulation of A. fumigatus germination were gained recently through a remarkable report by the research group of Joshua J. Obar published in *mSphere* (8).

**FIG 1:**
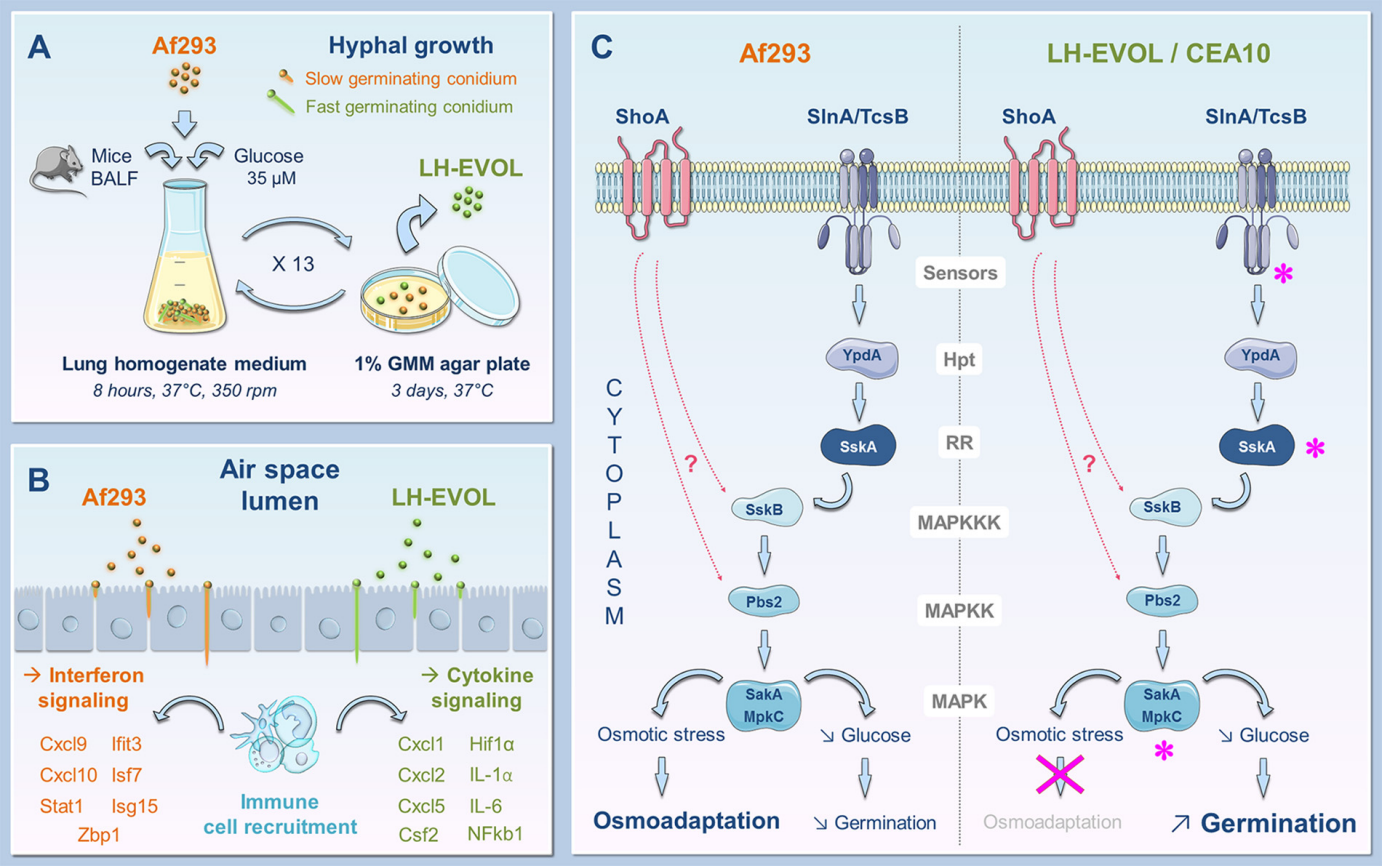
A. fumigatus germination in the airways is regulated by SskA through the SakA mitogen-activated protein kinase (MAPK) pathway and drives enhanced disease initiation and inflammation in the lungs. (A) By performing a serial passage approach of the Af293 reference strain (parental) in a murine-lung based medium, authors first selected a quick germinating strain (LH-EVOL) of A. fumigatus. (B) The LH-EVOL was able to induce inflammation *in vivo* at a greater extent compared to the parental strain with increased mRNA expression levels of interleukin 1α (IL-1α). (C) The stress-sensing pathway in A. fumigatus is composed of two main branches, i.e., ShoA and SlnA. Both branches were previously shown to modulate the so-called “high osmolarity glycerol (HOG) pathway,” a MAPK pathway that operates under stressing conditions. Upon diverse stressing conditions, the HOG pathway is activated and corresponds to the sequential phosphorylation of MAPKs involving SskB (MAPKKK), Pbs2 (MAPKK), and finally a couple of paralogous MAPK, i.e., SakA and MpkC. In this work, authors identified a loss-of-function allele of the *sskA* gene encoding a response regulator protein (RR) involved in the SlnA branch (also referred to as the two-component system). In line with this, they also showed that CEA10, a strain of A. fumigatus previously described for rapidly germinating both *in vitro* in lung homogenate medium and *in vivo* in murine lungs, displays mutations in both the *slnA/tcsB* and *mpkC* genes.

By performing a serial passage approach of the Af293 reference strain (parental) in a murine-lung based medium, Kirkland and colleagues first selected a quick germinating strain (LH-EVOL) of A. fumigatus (Fig. 1) (8). The LH-EVOL was able to induce inflammation *in vivo* at a greater extent compared with the parental strain with increased mRNA expression levels of interleukin 1α (IL-1α) (Fig. 1). Interestingly, the importance of this proinflammatory cytokine for host resistance against highly virulent strains of A. fumigatus was previously documented (6). In order to identify the genetic determinants underlying the increased germination rate observed in the LH-EVOL strain, the authors have performed a whole-genome variant analysis. By this way, they identified a loss-of-function allele of the *sskA* gene encoding a response regulator protein involved in the SlnA branch (also referred to as the two-component system, TCS) (9). More specifically, SskA protein was previously shown to modulate the so-called “high osmolarity glycerol (HOG) pathway,” a mitogen-activated protein kinase (MAPK) pathway that operates under stressing conditions in Saccharomyces cerevisiae (10) (Fig. 1). In this regard, several studies have demonstrated the pivotal role of the HOG signaling pathway in stress adaptation and virulence in prominent yeast pathogens such as Candida albicans and Cryptococcus neoformans, and also A. fumigatus (11–14). In these models and upon diverse stressing conditions, the HOG pathway is activated and corresponds to the sequential phosphorylation of MAPKs involving SskB (MAPKKK), Pbs2 (MAPKK), and finally a couple of paralogous MAPK, i.e., SakA and MpkC (13). In A. fumigatus, this phosphorylation cascade is regulated by two upstream stress-signaling pathways including SlnA (also referred to as TcsB) and the ShoA branches (15) (Fig. 1). In sum, these observations suggested that the *sskA* mutation detected in the LH-EVOL strain could be at the origin of an impaired regulation of the HOG pathway. In this respect, the SakA signaling response and stress tolerance were found to be decreased in a similar manner under osmotic stress in both LH-EVOL and Δ*sakA* mutant (the Af293 strain deleted for the *sakA* gene) when compared to the Af293 parental strain. In addition, deletion of genes encoding SskA, SakA, or MpkC in the parental strain Af293 was correlated with increased germination rates in both *in vitro* and *in vivo* assays. In line with this, Kirkland and colleagues showed that CEA10, a strain of A. fumigatus previously described for rapidly germinating both *in vitro* in lung homogenate medium and *in vivo* in murine lungs (6), displays mutations in both the *slnA/tcsB* and *mpkC* genes. Taken together, all these observations raise the idea that disruption of a cell signaling circuitry involving SskA, SlnA/TcsB, and MpkC may positively influence the germination process in A. fumigatus within the airways (Fig. 1). In an ultimate series of experiments, the authors nicely provide evidence that the low glucose availability in the lung prevents A. fumigatus germination in the lungs through SskA-SakA activation. Indeed, fungal mutant strains for effectors of this signaling pathway (i.e., *sskA*, *sakA*, and *mpkC*) were found to be predisposed to actively germinate under low glucose concentration *in vitro*.

Overall, this excellent report throws light on the central role of the HOG pathway regulation in governing the germination process in A. fumigatus in particular growth conditions such as those encountered in the airways. These data must be primarily compared to a recent study reporting the occurrence of missense mutations in the gene encoding the MAPKK (Pbs2) of the A. fumigatus HOG pathway in persistent strains recovered from the lungs of a CF patient (16). This key fact may seem intriguing at first glance because it is now well ingrained in the literature that this prominent MAPK cascade plays an essential role in the fungal adaptation to a broad range of environmental stresses. It is thus obviously appealing to consider that this pathogenic mold must sacrifice an important cell signaling circuitry to cope with the specific and long-term physicochemical constraints in the lungs. This physiological cost is evidenced by the fact that A. fumigatus strains disrupted for this signaling pathway display increased susceptibility to osmotic and oxidative stresses *in vitro*. Above all, this may indicate that persistent strains in the airways, i.e., isolates that develop the ability to chronically colonize the lungs, may drastically reconfigure their stress-sensing pathways for an adequate and long-term adaptation to the lung microenvironment. In such a perspective, mice models of acute and chronic aspergillosis should be considered in the near future to address this hypothesis.

In conclusion, this enlightening article teaches us once again how fungal pathogens can rapidly genetically evolve to dynamically adapt to specific niches. In this regard, the fungal genome plasticity now stands out as a major mechanism driving virulence and antifungal resistance regulation in pathogenic yeast and molds (17, 18). Such investigations must continue to potentially identify, in the near future, new therapeutic avenues to fight these life-threatening infectious diseases.
